# Efficiency and cost-effectiveness of three surgical strategies for primary osteoarthritis of the elbow: a multicenter propensity score–weighted cohort study

**DOI:** 10.1007/s00264-026-07001-z

**Published:** 2026-08-20

**Authors:** Shiyang Yu, Yanmao Wang, Shicheng Zhou, Fanhui Zeng, Jian Ding, Shengdi Lu

**Affiliations:** 1 Department of Orthopaedics, Shanghai Sixth People’s Hospital, Shanghai, China; 2 Department of Orthopaedics, Second Affiliated Hospital of Jilin University, Changchun, China; 3 Department of Orthopaedics, Nanchang Hongdu hospital of Traditional Chinese Medicine, Nanchang, China; 4 Pennington Biomedical Research Center, Baton Rouge, USA; 5 Department of Orthopaedics, Shanghai Sixth People’s Hospital, Shanghai, China

**Keywords:** Elbow osteoarthritis, Osteocapsular arthroplasty, Total elbow arthroplasty, Cost-effectiveness, Overlap weighting, Elbow contracture release

## Abstract

**Background:**

Primary osteoarthritis of the elbow causes progressive stiffness and pain, yet no study has directly compared the efficiency and cost-effectiveness of the three principal surgical strategies: total elbow arthroplasty (TEA), open osteocapsular arthroplasty (OCA), and arthroscopic OCA. We hypothesized that joint-preserving OCA would achieve greater motion recovery at lower cost than TEA.

**Methods:**

This multicenter retrospective cohort study identified 542 patients with primary elbow OA from three Chinese Tertiary A hospitals (surgery January 2017–December 2023; minimum two year follow-up through December 2025): 142 TEA, 221 open OCA, and 179 arthroscopic OCA. Because propensity-score matching left substantial residual imbalance in age and baseline motion, primary inference was based on generalized overlap weighting, which achieved covariate balance and estimates the average treatment effect in the overlap population, that is, the sub-population in clinical equipoise [25, 40]. Matching with covariate-adjusted mixed models is reported as a supporting analysis. Co-primary outcomes were the percentage of lost motion recovered and the flexion–extension (FE) arc at two years. Because pain relief rather than motion is the principal indication for TEA, cost-effectiveness was additionally assessed on a pain axis. Willingness-to-pay thresholds were not applied, as gross-domestic-product–based thresholds are defined per quality-adjusted life year and are not commensurate with effects expressed in natural units [4, 27].

**Results:**

Overlap weighting achieved acceptable balance (age standardized mean difference 0.11; baseline arc 0.08) with an effective sample size of 246 of 542 patients (45.4%; TEA 41, open OCA 144, arthroscopic OCA 62). In the overlap population, the percentage of lost motion recovered at 2 years was 15.8% (TEA), 35.9% (open OCA), and 32.2% (arthroscopic OCA), and the 2-year FE arc was 94.0°, 108.0°, and 104.5°. Both OCA procedures exceeded TEA by 16–20 percentage points of lost motion recovered, whereas the difference between the two OCA approaches was below the minimal clinically important difference. On the pain axis, visual analog scale reduction was 1.93, 2.44, and 2.77 points; all between-group differences (0.33–0.84) fell below the 1.4-point minimal clinically important difference, indicating clinically equivalent pain relief. Overlap-weighted total costs were ¥86,728 (TEA), ¥32,503 (open OCA), and ¥24,677 (arthroscopic OCA). TEA therefore cost approximately ¥62,000 more per patient than arthroscopic OCA without a clinically important advantage on either axis. Corrected complication rates in the TEA group were radiographic aseptic loosening 13.6%, loosening requiring revision 8.2%, and revision surgery 9.4%, consistent with published mid-term series.

**Conclusions:**

Within the sub-population in clinical equipoise, joint-preserving OCA recovered more motion than TEA at substantially lower cost, while pain relief was clinically equivalent across all three strategies. These findings describe real-world treatment strategies applied to their usual indications and should not be read as evidence that TEA and OCA are interchangeable, nor as a general first-line recommendation: patients with end-stage ulnohumeral destruction, for whom TEA is indicated, are under-represented in the overlap population. For patients in whom both joint preservation and arthroplasty are genuinely being considered, OCA appears the more efficient choice.

**Supplementary Information:**

The online version contains supplementary material available at https://doi.org/10.1007/s00264-026-07001-z.

## Background

Primary osteoarthritis (OA) of the elbow is an under-recognized condition. Unlike the hip and knee, the elbow was long considered resistant to degenerative wear. Morrey first described the entity in 1992, noting that it predominantly affects middle-aged men engaged in heavy manual labour [32]. Prevalence estimates remain scarce. Most series are small, retrospective, and drawn from single institutions. The condition nonetheless causes substantial disability. Patients develop painful stiffness, mechanical locking from loose bodies, and progressive loss of the functional arc of motion, defined by Morrey et al. as 30° to 130° of flexion–extension [31].

Three broad surgical strategies exist [7, 21]. Joint-preserving osteocapsular arthroplasty (OCA), performed either through an open or an arthroscopic approach, aims to restore motion by excising osteophytes, removing loose bodies, and releasing the contracted capsule. Antuña et al. reported durable gains with open ulnohumeral arthroplasty at seven year follow-up [2]. Kelly et al. demonstrated comparable improvements via arthroscopy [17]. A systematic review by de Klerk et al. found that both approaches yield meaningful gains in range of motion and pain relief, but direct comparative data remain limited [8]. Total elbow arthroplasty (TEA) is reserved for end-stage disease and is undertaken principally to relieve pain rather than to restore motion; Welsink et al. found that roughly 60% of patients were pain-free at mean 6.3 years, while extension lag averaged 30 [44], and Martínez-Catalán and Sanchez-Sotelo describe TEA as a salvage option for older, lower-demand patients that provides reliable pain relief but inconsistent motion gain and requires lifelong activity restriction [28]. Schoch et al. reported reliable pain relief but inconsistent extension recovery after TEA for primary OA [38]. Parker et al. confirmed high complication rates in a broader TEA systematic review [34].

A critical gap persists in the literature. No study has directly compared the efficiency and cost-effectiveness of all three surgical strategies, TEA, open OCA, and arthroscopic OCA, within a single cohort. Existing evidence is fragmented across single-procedure case series, most with small samples and no economic analysis. This gap is especially acute in China, where rising healthcare expenditure and the national adoption of diagnosis-related group (DRG) payment reform demand rigorous cost-effectiveness data to guide surgical decision-making.

The purpose of this study was to compare the efficiency and cost-effectiveness of TEA, open OCA, and arthroscopic OCA for primary elbow OA in a multicenter Chinese cohort. We hypothesized that joint-preserving OCA procedures would achieve greater motion recovery at lower cost compared with TEA. Because the three procedures serve overlapping but distinct indications, we framed the comparison around the sub-population in which more than one strategy could reasonably be offered.

## Methods

### Study design and setting

This multicenter retrospective cohort study was conducted across three Chinese Tertiary A hospitals. Patients underwent surgery between January 2017 and December 2023, and all included patients completed a minimum of two years of follow-up by the December 2025 data-lock. Reporting followed the Strengthening the Reporting of Observational Studies in Epidemiology (STROBE) guidelines [43] and, for the economic component, the Consolidated Health Economic Evaluation Reporting Standards (CHEERS) 2022 statement [15]. Institutional review board approval was obtained at Shanghai Sixth People’s Hospital; informed consent was waived owing to the retrospective design. Detailed eligibility criteria, data collection procedures, and all medical codes used for electronic health record (EHR) extraction are provided in the Supplementary Material (Supplementary Table 1).

### Participants

Eligible patients were adults (≥ 18 years) with a primary discharge diagnosis of primary elbow OA (ICD-10 code M19.002 per GB/T 14396–2016) who underwent one of the three index procedures and had a minimum 2-year follow-up. Exclusion criteria included inflammatory arthropathy, post-traumatic OA, prior ipsilateral elbow arthroplasty, and insufficient follow-up. Full criteria are listed in the Supplementary Materials.

Treatment selection was not randomized. In accordance with prevailing practice at the participating centers, joint-preserving OCA was offered to patients with symptomatic osteophyte impingement, loose bodies, or a mechanical block to motion who retained a congruent ulnohumeral joint, whereas TEA was reserved for older, lower-demand patients with advanced ulnohumeral destruction, global joint-space loss, or failed prior joint-preserving surgery. The choice between an open and an arthroscopic approach to OCA was made at surgeon discretion. This indication-driven allocation is a central consideration in interpreting our findings and directly motivates our choice of estimand, described below.

### Procedures and outcomes

Patients were classified by index procedure: TEA (ICD-9-CM-3 code 81.8400), open OCA (80.4200/80.8200), or arthroscopic OCA (80.4202/80.8202). Both OCA approaches followed the same osteocapsular principles, anterior and posterior osteophyte excision, loose-body removal, fenestration or limited fossa recession where indicated, and capsular release, without prosthetic resurfacing or interposition arthroplasty. All TEA procedures used a linked (semiconstrained) Coonrad-Morrey–type prosthesis [14, 23]. Concomitant ulnar nerve decompression or transposition was performed when preoperative ulnar neuropathy was present (24%–36% of patients per group) and was included among the propensity-score covariates. Procedures were performed by fellowship-trained elbow or upper-extremity surgeons at high-volume tertiary centres; because outcomes of elbow arthroscopy are sensitive to surgeon experience [18], all arthroscopic OCA procedures were performed by surgeons beyond the reported learning-curve threshold.

The co-primary outcomes were the percentage of lost motion recovered at two years, calculated as [(postoperative arc − preoperative arc)/(145° − preoperative arc)] × 100% [5, 33], and the flexion–extension (FE) arc at two years. Secondary outcomes included the FE arc change, American Shoulder and Elbow Surgeons (ASES) elbow score change, and visual analog scale (VAS) pain with activity change at six weeks, three months, six months, one year, and two years. Because pain relief rather than motion restoration is the principal therapeutic goal of TEA [28, 38, 44], VAS pain reduction was prespecified as a second effectiveness axis for the economic analysis, so that each strategy could also be judged against the objective for which it is actually performed. Cost-effectiveness was assessed from the healthcare-system perspective using total cumulative direct medical costs in Chinese Yuan.

### Complication ascertainment

Complications were abstracted from operative notes, discharge summaries, radiology reports, and outpatient records, and were classified a priori as universal adverse events (applicable to all three procedures) or procedure-specific complications. Radiographic aseptic loosening was defined as a progressive radiolucent line ≥ 2 mm around any component or component migration on serial radiographs, irrespective of symptoms; loosening requiring revision was defined as radiographic loosening that led to an actual revision procedure. Conversion to open surgery denoted an intraoperative decision during an arthroscopic procedure and was distinguished from later conversion to TEA, which was recorded separately as a reintervention. Because complications are not amenable to imputation, all complication rates are reported as complete-case proportions with the number of patients with available data as the explicit denominator; denominators differ between rows because ascertainment completeness varied by data source. A composite of any surgical reintervention (reoperation, revision, or later conversion to TEA) was computed as the union of these events.

### Statistical analysis

Generalized propensity scores (GPS) were estimated by multinomial logistic regression on 15 baseline covariates [10, 16, 29]. Because the three treatment populations were strongly separated, greedy 1:1:1 matching on the GPS vector reduced but did not eliminate imbalance, leaving standardized mean differences (SMD) of 1.256 for age and 0.907 for baseline arc, far above the 0.10 target [3]. Covariate adjustment within such a sample would require extrapolation across regions where the groups scarcely coexist. We therefore designated generalized overlap weighting as the primary analysis [24]. Overlap weights are bounded between 0 and 1, achieve exact mean balance, minimize the asymptotic variance of the weighted estimator, and are robust to extreme propensity scores [25]. The resulting estimand is the average treatment effect in the overlap population (ATO), that is, the sub-population of patients for whom more than one strategy could plausibly be offered and who would be candidates for a pragmatic randomized trial [40]. We report the effective sample size (ESS), computed within each treatment group as the square of the sum of weights divided by the sum of squared weights, to quantify how much of the cohort contributes to this estimate. The matched cohort and its covariate-adjusted mixed models are retained as supporting analyses, and inverse probability of treatment weighting, full intention-to-treat, per-protocol, and complete-case analyses as sensitivity analyses. Missing data for continuous outcomes were handled by multiple imputation (m = 5) following a nonsignificant Little’s MCAR test (*P* > 0.99); complications were not imputed.

Unadjusted outcomes were compared using one-way analysis of variance. Supporting adjusted analyses used linear mixed-effects models with fixed effects for treatment, time, the treatment × time interaction, age, sex, and the baseline outcome value, and a patient-level random intercept; a doubly-robust estimator combining overlap weighting with covariate adjustment was also fitted [12]. Bonferroni correction was applied across three secondary outcome families. Incremental cost-effectiveness ratios (ICERs) were calculated with arthroscopic OCA as reference on two effectiveness axes, percentage of lost motion recovered and VAS pain reduction, using overlap-weighted costs and effects, with uncertainty quantified by 1,000 nonparametric bootstrap replicates. We deliberately did not compare ICERs against willingness-to-pay thresholds. Thresholds expressed as multiples of gross domestic product per capita are defined per disability-adjusted life year or per quality-adjusted life year [4, 27, 46], and applying them to effects measured in degrees of motion or points of pain would be a mismatch of units. Because we did not collect utility data, no quality-adjusted life years could be derived. We therefore report incremental costs and effects in natural units, together with dominance where one strategy is both less costly and more effective, a conclusion that is valid independently of any threshold [9], and cost-effectiveness planes showing the joint distribution of incremental cost and effect [11]. All differences are additionally interpreted against published minimal clinically important difference (MCID) thresholds for the elbow: 14.1° (distribution-based) and 25° (anchor-based) for range of motion and 12.2 points for the Mayo Elbow Performance Index [47], and 20.9 points for the ASES score and 1.4 points for VAS pain [39]. Prespecified subgroup analyses examined treatment effects by age, sex, preoperative arc (< 80° vs. ≥ 80° [26]), and occupation. Details are in the Supplementary Materials.

## Results

### Study population and the overlap population

**Fig. 1:**
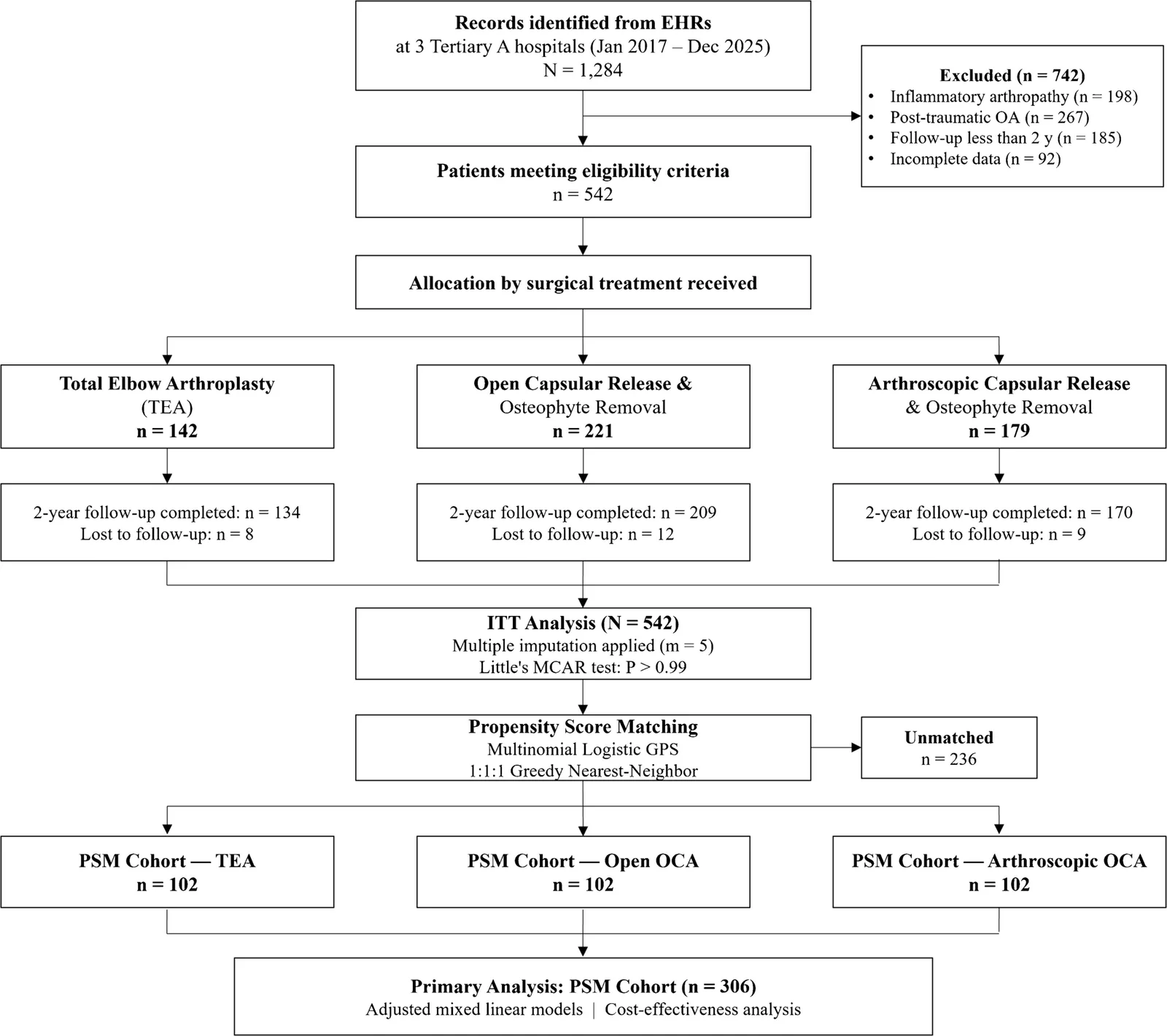
Study flow diagram. Of 1,284 records screened from 3 Chinese Tertiary A hospitals (surgery January 2017–December 2023; follow-up through December 2025), 742 were excluded, yielding 542 eligible patients allocated to TEA (*n* = 142), open OCA (*n* = 221), and arthroscopic OCA (*n* = 179). Primary analyses applied generalized overlap weighting to all 542 patients (effective sample size 246); propensity-score matching yielding 102 patients per group is reported as a supporting analysis

Of 1,284 records screened, 742 were excluded (Fig. 1). The remaining 542 patients comprised 142 TEA, 221 open OCA, and 179 arthroscopic OCA. Mean follow-up was 4.2 years (range, 2–7).

Overlap weighting achieved acceptable balance on the two principal confounders, reducing the maximum pairwise SMD for age from 1.929 to 0.109 and for baseline FE arc from 1.513 to 0.079; the maximum SMD across all 15 covariates was 0.348 (Supplementary Table 7). The effective sample size was 246 of 542 patients (45.4%), distributed as 41 of 142 in the TEA group (29%), 144 of 221 in the open OCA group (65%), and 62 of 179 in the arthroscopic OCA group (34%). The overlap population is therefore weighted towards patients in whom joint preservation and arthroplasty were both plausible options, and it under-represents the oldest, stiffest TEA recipients with end-stage destruction, for whom no realistic alternative existed. All primary estimates below describe this equipoise sub-population. For reference, propensity-score matching yielded 102 patients per group but left substantial residual imbalance (SMD 1.256 for age and 0.907 for baseline arc; Table 1, Supplementary Tables 2 and 3); matched results are reported as supporting analyses.

**Table 1:** Baseline characteristics in the matched supporting cohort (*n* = 102 per Group)

Variable	TEA (*n* = 102)	Open OCA (*n* = 102)	Arthroscopic OCA (*n* = 102)	*P* Value
Demographics				
Age, y, mean (SD)	60.3 (6.8)	56.1 (5.6)	52.6 (5.5)	< 0.001
Male sex, No. (%)	77 (75.5%)	74 (72.5%)	76 (74.5%)	0.484
BMI, kg/m^2^, mean (SD)	24.3 (3.0)	24.4 (2.6)	25.3 (2.6)	0.021
Current smoker, No. (%)	40 (39.2%)	45 (44.1%)	49 (48.0%)	0.470
Dominant arm involved, No. (%)	62 (60.8%)	64 (62.7%)	71 (69.6%)	0.479
Manual labor, No. (%)	53 (52.0%)	62 (60.8%)	50 (49.0%)	0.543
Clinical History				
Symptom duration, mo	31.2 (20.1)	33.2 (27.7)	29.0 (17.6)	0.412
Mechanical symptoms, No. (%)	25 (24.5%)	27 (26.5%)	20 (19.6%)	0.468
Ulnar nerve symptoms, No. (%)	33 (32.4%)	37 (36.3%)	24 (23.5%)	0.450
Steroid injections, mean (SD)	0.8 (0.9)	0.7 (0.8)	0.8 (0.9)	0.825
Prior surgery, No. (%)	20 (19.6%)	8 (7.8%)	8 (7.8%)	0.287
Comorbidities				
Diabetes (T2), No. (%)	16 (15.7%)	13 (12.7%)	11 (10.8%)	0.549
CVD, No. (%)	47 (46.1%)	44 (43.1%)	15 (14.7%)	0.033
ASA I	19 (18.6%)	20 (19.6%)	24 (23.5%)	0.455
ASA II	44 (43.1%)	55 (53.9%)	49 (48.0%)	0.455
ASA III	19 (18.6%)	11 (10.8%)	11 (10.8%)	0.455
Preoperative Measures				
Flexion, °	111.1 (7.8)	113.8 (7.0)	115.8 (8.4)	< 0.001
Extension deficit, °	29.2 (6.9)	27.4 (6.9)	23.7 (5.6)	< 0.001
FE arc, °	81.7 (11.4)	85.3 (9.8)	91.8 (10.8)	< 0.001
Pronation, °	69.7 (6.0)	71.4 (6.2)	73.7 (5.0)	< 0.001
Supination, °	65.6 (8.2)	68.4 (7.0)	68.3 (7.9)	0.015
ASES score	43.0 (10.5)	45.9 (9.3)	49.7 (9.1)	< 0.001
VAS rest	3.6 (1.1)	3.1 (0.9)	2.9 (0.9)	< 0.001
VAS activity	6.1 (1.4)	5.7 (1.3)	5.4 (1.0)	< 0.001
Surgical				
Operative time, min	143.9 (35.8)	103.9 (23.2)	86.2 (24.3)	< 0.001
LOS, d	9.0 (2.8)	6.8 (1.7)	4.8 (1.9)	< 0.001

### Primary outcomes

**Fig. 2:**
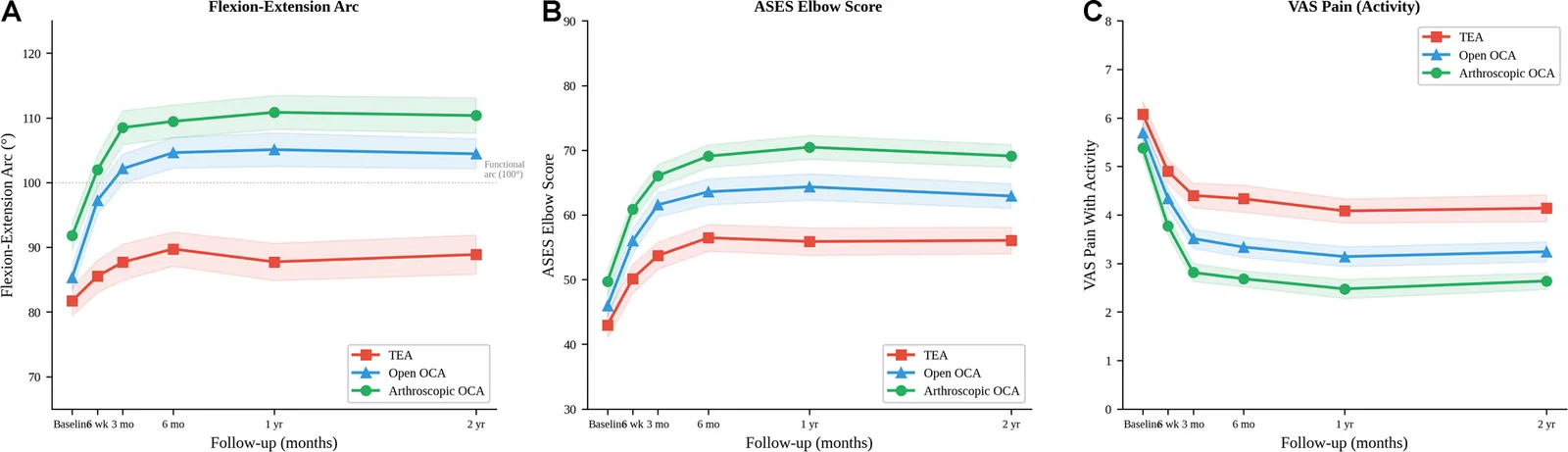
Adjusted recovery trajectories in the matched supporting cohort (*n *= 102 per group). Panel **A**: FE arc (°). Panel **B**: ASES elbow score. Panel **C**: VAS pain with activity. Lines represent group means; shaded bands represent 95% confidence intervals. The dashed line in Panel **A** indicates the 100° functional arc threshold

In the overlap population, the percentage of lost motion recovered at two years was 15.8% for TEA, 35.9% for open OCA, and 32.2% for arthroscopic OCA, and the two year FE arc was 94.0°, 108.0°, and 104.5°, respectively (Supplementary Table 7). Both OCA procedures exceeded TEA by a wide margin (open OCA + 20.1 percentage points; arthroscopic OCA + 16.5), and a doubly-robust estimator combining overlap weighting with covariate adjustment gave near-identical results (+ 20.1 and + 16.4). The corresponding FE arc advantages over TEA (14.0° for open and 10.5° for arthroscopic OCA) approached but did not uniformly exceed the 14.1° distribution-based MCID, and neither reached the 25° anchor-based threshold [47]; the difference between the two OCA approaches (3.5°) was well below both. In the supporting matched analysis, the percentage of lost motion recovered was 12.9% ± 13.6% for TEA, 34.6% ± 15.6% for open OCA, and 37.2% ± 18.1% for arthroscopic OCA (*P* < 0.001; Table 2), the 2-year FE arc was 87.9° ± 15.0°, 105.2° ± 12.9°, and 109.6° ± 13.4° (*P* < 0.001), and the treatment × time interaction was significant for all outcome domains (*P* < 0.001; Table 3, Fig. 2).

**Table 2:** Unadjusted outcomes in the matched supporting cohort

Outcome	TEA Mean (SD)	Open OCA Mean (SD)	Arthroscopic OCA Mean (SD)	*P* Value
Primary Outcomes				
% Lost motion recovered at 2y	12.9 (13.6)	34.6 (15.6)	37.2 (18.1)	< 0.001
FE arc at 2y, °	87.9 (15.0)	105.2 (12.9)	109.6 (13.4)	< 0.001
Secondary Outcomes: Range of Motion				
FE arc change, 6 Weeks	3.8 (11.2)	11.9 (8.7)	10.2 (9.6)	< 0.001
FE arc change, 3 Months	6.0 (12.3)	16.8 (10.5)	16.7 (10.5)	< 0.001
FE arc change, 6 Months	8.0 (12.9)	19.3 (11.7)	17.7 (10.9)	< 0.001
FE arc change, 1 Year	6.0 (11.8)	19.8 (10.0)	19.1 (11.7)	< 0.001
FE arc change, 2 Years	7.2 (12.2)	19.1 (10.6)	18.6 (11.2)	< 0.001
Secondary Outcomes: Functional Scores				
ASES change, 6 Weeks	7.2 (7.6)	10.1 (7.3)	11.2 (8.2)	< 0.001
ASES change, 3 Months	10.7 (9.8)	15.6 (8.3)	16.4 (8.1)	< 0.001
ASES change, 6 Months	13.5 (9.6)	17.6 (8.0)	19.4 (8.7)	< 0.001
ASES change, 1 Year	12.9 (9.8)	18.4 (8.6)	20.8 (8.8)	< 0.001
ASES change, 2 Years	13.1 (9.6)	17.0 (8.8)	19.4 (8.7)	< 0.001
VAS activity change, 6 Weeks	−1.2 (1.0)	−1.4 (0.7)	−1.6 (0.8)	0.006
VAS activity change, 3 Months	−1.7 (1.2)	−2.2 (1.0)	−2.6 (1.0)	< 0.001
VAS activity change, 6 Months	−1.7 (1.3)	−2.4 (1.2)	−2.7 (1.1)	< 0.001
VAS activity change, 1 Year	−2.0 (1.2)	−2.5 (1.1)	−2.9 (1.0)	< 0.001
VAS activity change, 2 Years	−1.9 (1.1)	−2.4 (1.0)	−2.7 (1.1)	< 0.001

**Table 3:** Covariate-adjusted outcomes from linear mixed-effects models (Matched Supporting Cohort)

Outcome	TEA	Open OCA	Arthroscopic OCA	P Value	Covariates
FE arc, ° (Adjusted)					
6 Weeks	85.5	97.2	102.0	< 0.001	Baseline Characteristics
3 Months	87.7	102.1	108.5	< 0.001	Baseline Characteristics
6 Months	89.7	104.6	109.5	< 0.001	Baseline Characteristics
1 Year	87.7	105.1	110.9	< 0.001	Baseline Characteristics
2 Years	88.9	104.4	110.4	< 0.001	Baseline Characteristics
Treatment × Time P				< 0.001	
ASES score (Adjusted)					
6 Weeks	50.2	56.0	60.9	< 0.001	Baseline Characteristics
3 Months	53.7	61.6	66.1	< 0.001	Baseline Characteristics
6 Months	56.5	63.6	69.1	< 0.001	Baseline Characteristics
1 Year	55.9	64.3	70.5	< 0.001	Baseline Characteristics
2 Years	56.1	62.9	69.1	< 0.001	Baseline Characteristics
Treatment × Time P				0.017	
VAS activity (Adjusted)					
6 Weeks	4.9	4.3	3.8	< 0.001	Baseline Characteristics
3 Months	4.4	3.5	2.8	< 0.001	Baseline Characteristics
6 Months	4.3	3.3	2.7	< 0.001	Baseline Characteristics
1 Year	4.1	3.1	2.5	< 0.001	Baseline Characteristics
2 Years	4.1	3.2	2.6	< 0.001	Baseline Characteristics
Treatment × Time P				0.021

### Pain relief

On the pain axis, overlap-weighted VAS pain reduction at two years was 1.93 points for TEA, 2.44 for open OCA, and 2.77 for arthroscopic OCA. Every between-group difference (open versus TEA + 0.51; arthroscopic versus TEA + 0.84; arthroscopic versus open + 0.33) fell below the 1.4-point MCID for VAS pain [39], indicating that the three strategies delivered clinically equivalent pain relief. Overlap-weighted ASES improvement was 13.1, 17.3, and 21.0 points respectively; the largest between-group difference (7.9 points) likewise remained below the 20.9-point ASES MCID [39]. Thus, on the outcome for which TEA is principally undertaken, no strategy demonstrated a clinically important advantage.

### Adverse events and complications

Complication rates, reported as complete-case proportions with explicit denominators, are shown in Table 4. Universal adverse events did not differ significantly across groups: superficial wound infection 1.1%–2.2%, deep infection 0%–3.3%, symptomatic venous thromboembolism 0%–3.5%, heterotopic ossification 1.2%–3.6%, and any reoperation 4.9% (TEA), 15.1% (open OCA), and 8.9% (arthroscopic OCA) (all P ≥ 0.086). A composite of any surgical reintervention occurred in 11.9%, 15.8%, and 9.0% respectively (*P* = 0.332). In the TEA group, radiographic aseptic loosening was observed in 13.6% (11 of 81), loosening requiring revision in 8.2% (7 of 85), revision surgery in 9.4% (8 of 85), ulnar neuropathy in 4.4% (4 of 90; 3.3% transient, 1.1% permanent), triceps insufficiency in 2.2% (2 of 92), and implant dislocation in 1.1% (1 of 89). Open OCA was associated with ulnar nerve injury in 16.9% (15 of 89; 10.1% transient, 6.7% permanent), recurrent stiffness in 16.7% (14 of 84), wound haematoma in 1.2% (1 of 84), and later conversion to TEA in 2.4% (2 of 84). Arthroscopic OCA was associated with ulnar nerve injury in 5.7% (5 of 87; 2.3% transient, 3.4% permanent), persistent portal drainage in 7.2% (6 of 83), portal complications in 4.8% (4 of 84), intraoperative conversion to an open procedure in 2.4% (2 of 83), and later conversion to TEA in 1.2% (1 of 85). These figures were reproduced in the full 542-patient cohort (TEA loosening requiring revision 8.3%; arthroscopic conversion to open 2.0%).

**Table 4:** Adverse events and complications by treatment group

Adverse Event	TEA	Open OCA	Arthroscopic OCA	*P* Value
Universal adverse events, No./n (%)				
Superficial wound infection	1/88 (1.1)	2/97 (2.1)	2/93 (2.2)	0.892
Deep infection	3/91 (3.3)	0/93 (0.0)	1/89 (1.1)	0.270
Heterotopic ossification	1/82 (1.2)	3/83 (3.6)	1/84 (1.2)	0.532
Symptomatic VTE (DVT/PE)	3/86 (3.5)	0/87 (0.0)	1/85 (1.2)	0.271
Any reoperation	4/82 (4.9)	14/93 (15.1)	8/90 (8.9)	0.086
Any surgical reintervention (composite)	12/101 (11.9)	16/101 (15.8)	9/100 (9.0)	0.332
TEA-specific complications, No./n (%)				
Aseptic loosening, radiographic	11/81 (13.6)	—	—	
Aseptic loosening requiring revision	7/85 (8.2)	—	—	
Revision surgery	8/85 (9.4)	—	—	
Ulnar neuropathy (3.3% transient, 1.1% permanent)	4/90 (4.4)	—	—	
Triceps insufficiency	2/92 (2.2)	—	—	
Implant dislocation	1/89 (1.1)	—	—	
Open OCA-specific complications, No./n (%)				
Ulnar nerve injury (10.1% transient, 6.7% permanent)	—	15/89 (16.9)	—	
Recurrent stiffness	—	14/84 (16.7)	—	
Wound hematoma	—	1/84 (1.2)	—	
Later conversion to TEA	—	2/84 (2.4)	—	
Arthroscopic OCA-specific complications, No./n (%)				
Ulnar nerve injury (2.3% transient, 3.4% permanent)	—	—	5/87 (5.7)	
Persistent portal drainage	—	—	6/83 (7.2)	
Portal complications	—	—	4/84 (4.8)	
Intraoperative conversion to open	—	—	2/83 (2.4)	
Later conversion to TEA	—	—	1/85 (1.2)	

Data are number of events/number of patients with available data (percentage). Because complications are not amenable to imputation, all rates are complete-case proportions and denominators differ between rows according to ascertainment completeness. P values are from chi-square or Fisher exact tests for universal adverse events; procedure-specific complications are descriptive. The composite of any surgical reintervention is the union of reoperation, revision, and later conversion to TEA. Radiographic aseptic loosening denotes a progressive radiolucent line ≥ 2 mm or component migration irrespective of symptoms. — = not applicable. *VTE* venous thromboembolism, *DVT* deep vein thrombosis, *PE* pulmonary embolism

### Cost-effectiveness

**Fig. 3:**
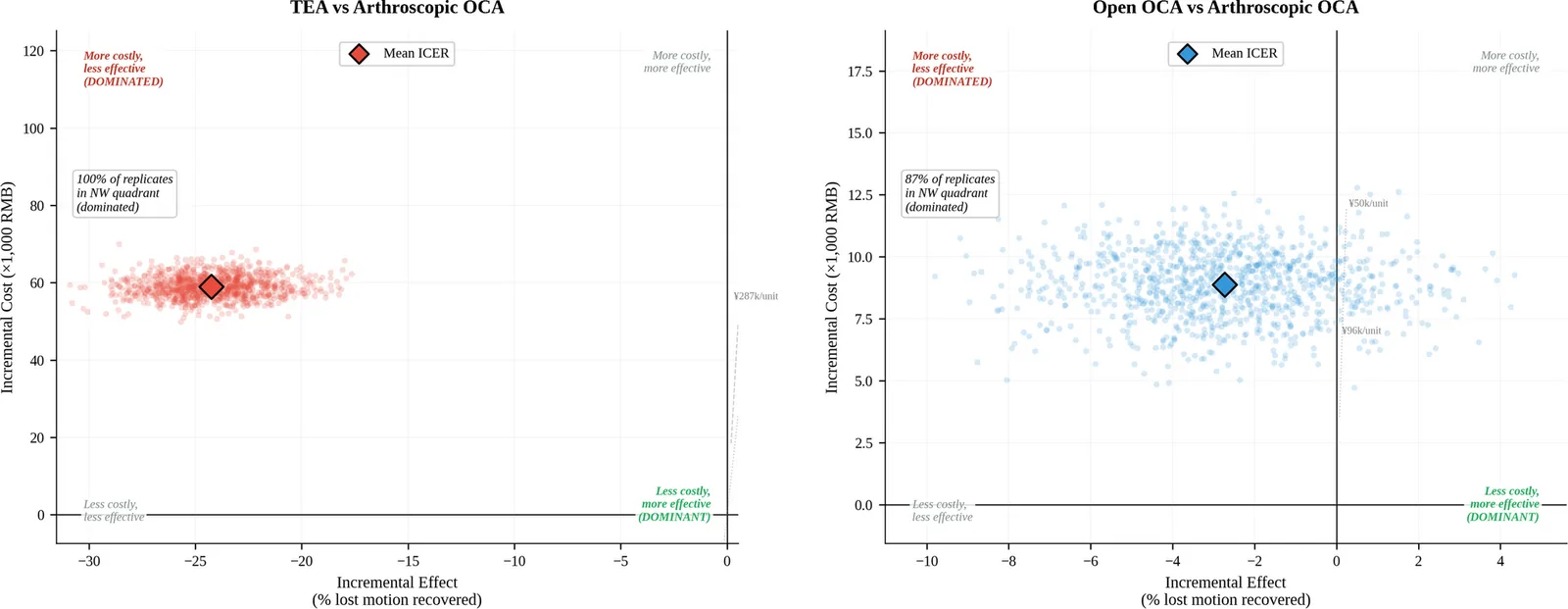
Cost-effectiveness planes from 1,000 nonparametric bootstrap replicates using overlap-weighted costs and effects. Upper row: percentage of lost motion recovered. Lower row: VAS pain reduction. Left panels: TEA versus arthroscopic OCA. Right panels: open OCA versus arthroscopic OCA. No willingness-to-pay threshold lines are shown, because thresholds derived from gross domestic product per capita are defined per quality-adjusted life year and are not commensurate with effects in natural units

Overlap-weighted total cumulative costs were ¥86,728 for TEA, ¥32,503 for open OCA, and ¥24,677 for arthroscopic OCA; corresponding matched-cohort costs are shown in Table 5. Relative to arthroscopic OCA, TEA cost ¥62,050 more per patient while recovering 16.5 percentage points less lost motion and achieving 0.84 points less pain reduction, and open OCA cost ¥7,826 more while recovering 3.6 percentage points more lost motion and 0.33 points less pain reduction (Table 6). TEA was thus strictly dominated by arthroscopic OCA on both axes, a conclusion that does not depend on any willingness-to-pay threshold [9]. This formal dominance should nonetheless be read with care: on the pain axis the effectiveness difference underlying it (0.84 points) is below the MCID, so the economically meaningful statement is that TEA cost substantially more without delivering clinically superior pain relief, rather than that it was less effective in any clinically perceptible sense. The comparison between the two OCA approaches is likewise driven by cost rather than effectiveness, since both effect differences lie below their respective MCIDs. Cost-effectiveness planes are shown in Fig. 3.

**Table 5:** Direct medical costs (Matched Supporting Cohort, Chinese Yuan)

Cost (Chinese Yuan)	TEA	Open OCA	Arthroscopic OCA	*P* Value
Surgical fees	4466.6 (783.1)	3504.1 (449.7)	3036.3 (504.2)	< 0.001
Implant costs	48,359.2 (19,154.2)	9960.2 (5864.7)	5471.6 (5989.3)	< 0.001
Anesthesia	3309.9 (806.5)	2175.2 (472.1)	1917.2 (408.0)	< 0.001
Room/bed	3174.8 (1168.9)	2410.5 (830.1)	1752.6 (802.4)	< 0.001
Nursing	1119.8 (494.8)	827.1 (283.2)	563.0 (274.7)	< 0.001
Lab	2365.4 (570.8)	1818.2 (422.5)	1552.6 (306.3)	< 0.001
Imaging	1774.6 (477.9)	1186.0 (378.9)	1055.7 (308.0)	< 0.001
Pharmacy	8353.6 (2928.9)	5483.1 (1853.6)	4512.0 (1585.4)	< 0.001
Materials	2020.2 (646.0)	1561.7 (444.2)	1195.3 (412.1)	< 0.001
Rehab	1507.3 (563.2)	1217.0 (463.9)	851.3 (388.6)	< 0.001
Blood transfusion	100.4 (357.6)	65.9 (243.7)	6.0 (17.7)	0.025
Total index hospitalization	77,957.1 (20,482.7)	30,142.5 (6730.3)	22,371.0 (8142.6)	< 0.001
LOS, days	9.0 (2.8)	6.8 (1.7)	4.8 (1.9)	< 0.001
90-day readmission rate	2 (2.0%)	2 (2.0%)	1 (1.0%)	0.505
Total cumulative cost	82,533.9 (27,399.9)	32,602.3 (9453.7)	23,705.1 (9833.0)	< 0.001

**Table 6:** Cost-effectiveness analysis in the overlap population on two effectiveness axes

Strategy	Overlap-weighted cost (¥)	Incremental cost vs. reference (¥)	Effect	Incremental effect	Interpretation
Axis 1: Percentage of lost motion recovered at 2 years					
TEA	86,728	+ 62,050	15.8%	− 16.5 pp	Dominated
Open OCA	32,503	+ 7,826	35.9%	+ 3.6 pp	¥2,174 per additional pp
Arthroscopic OCA (reference)	24,677	Ref	32.2%	Ref	Reference
Axis 2: VAS pain reduction at 2 years					
TEA	86,728	+ 62,050	1.93 pts	− 0.84 pts	Dominated (below MCID)
Open OCA	32,503	+ 7,826	2.44 pts	− 0.33 pts	Dominated (below MCID)
Arthroscopic OCA (reference)	24,677	Ref	2.77 pts	Ref	Reference

Costs and effects are overlap-weighted and therefore describe the overlap (equipoise) population (effective sample size 246 of 542). Arthroscopic OCA is the reference strategy because it was least costly. A strategy is dominated when it is both more costly and less effective; dominance is a valid conclusion independently of any willingness-to-pay threshold. No willingness-to-pay threshold is applied in this analysis: thresholds expressed as multiples of gross domestic product per capita are defined per quality-adjusted or disability-adjusted life year and are not commensurate with effects expressed in natural units, and no utility data were collected. Differences on both axes should be interpreted against minimal clinically important differences (14.1° distribution-based for motion; 1.4 points for VAS pain); where a dominance verdict rests on a difference below the MCID this is stated explicitly. *pp* percentage points, *pts* points, *MCID* minimal clinically important difference

### Sensitivity and subgroup analyses

All sensitivity analyses were concordant with the primary overlap-weighted result (Supplementary Table 5, Supplementary Fig. 2). The full intention-to-treat analysis using all 542 patients yielded effect sizes at least as large as the matched analysis (percentage of lost motion recovered: TEA 11.8%, open OCA 38.0%, arthroscopic OCA 42.0%; *P* < 0.001). Inverse probability of treatment weighting, which improved but did not fully achieve balance (age SMD 0.744), gave contrasts of + 24.4 and + 22.4 percentage points versus TEA. Per-protocol and complete-case analyses each yielded *P* < 0.001 for both co-primary outcomes. Subgroup analyses showed consistent OCA advantage across all strata of age, sex, preoperative arc, and occupation, with no qualitative interactions (Supplementary Table 6, Supplementary Fig. 3).

## Discussion

This multicenter study provides the first three-way comparison of TEA, open OCA, and arthroscopic OCA for primary elbow OA. Four findings emerged. First, within the sub-population in clinical equipoise, joint-preserving procedures recovered substantially more motion than TEA. Second, pain relief was clinically equivalent across all three strategies. Third, TEA cost approximately ¥62,000 more per patient than arthroscopic OCA without a clinically important advantage on either effectiveness axis. Fourth, the difference between the two OCA approaches was economic rather than clinical.

The estimand deserves emphasis. Overlap weighting estimates the effect in the population where the propensity distributions of the three strategies genuinely overlap, which is the population a pragmatic randomized trial could enroll [25, 40]. Our effective sample size of 246 of 542 patients, and particularly of only 41 of 142 TEA recipients, quantifies how narrow that population is. Patients with end-stage ulnohumeral destruction, for whom TEA is the only realistic option, contribute little weight and are not represented by these estimates. Our results should therefore not be read as a general first-line recommendation for elbow OA, and still less as evidence that TEA is an inferior operation. They describe the narrower and more useful question of what happens when a surgeon is genuinely choosing between joint preservation and arthroplasty for the same patient.

The choice of effectiveness axis matters for the same reason. TEA is undertaken in end-stage joints to relieve pain, not to restore motion [28, 38, 44], so judging it solely on motion recovered would measure it against an objective it does not pursue. We therefore analysed pain as a co-equal axis. On that axis all three strategies were clinically equivalent, every between-group difference falling below the 1.4-point VAS MCID [39]. This strengthens rather than weakens the economic argument, because it shows that the cost differential is not purchasing additional pain relief. It also tempers the motion-based comparison: TEA achieved its own therapeutic goal about as well as the alternatives, at considerably greater cost, in the equipoise population.

We also reconsidered our economic framing. Our earlier analysis compared ICERs expressed per unit of motion against willingness-to-pay thresholds derived from gross domestic product per capita. Those thresholds are defined per disability-adjusted or quality-adjusted life year [4, 27], including in the Chinese pharmacoeconomic guidelines [46], and applying them to a per-degree effect mismatches the units and cannot support a formal verdict of cost-effectiveness. Because we collected no utility data, we could not construct quality-adjusted life years, the reference-case effectiveness metric [37]. We have therefore removed the threshold comparison entirely and confined our economic claims to incremental costs and effects in natural units, to dominance where it holds, which requires no threshold [9], and to the joint distribution of cost and effect [11]. We report the analysis in accordance with CHEERS 2022 [15]. A cost-utility analysis using a preference-based instrument remains necessary before any formal statement about value for money can be made.

A further revision concerns the complication data. On re-auditing our source records we identified an error in the original tabulation, in which structurally missing procedure-specific fields had been counted against a fixed denominator of 102 per group, inflating every procedure-specific rate. The corrected complete-case figures are materially lower and align with the published literature. Our TEA radiographic loosening rate of 13.6% and revision-for-loosening rate of 8.2% sit close to the 17.2% and 6.3% reported by Parker et al. in their systematic review of contemporary TEA [34]; our ulnar neuropathy rate of 4.4% and triceps insufficiency rate of 2.2% are below the 9% and 7% reported by Toulemonde et al. in 100 semiconstrained TEAs at mean five years [41]; and our revision rate is consistent with Norwegian Arthroplasty Register survivorship of 92% at five years [20]. Our arthroscopic conversion-to-open rate of 2.4% and reoperation rate of 8.9% fall within the 1.5–11% complication and up-to-11.8% reoperation ranges reported by Tsenkov and Dimitrov [42]. One figure warrants explicit comment: our arthroscopic ulnar nerve injury rate of 5.7% exceeds the pooled arthroscopic neurologic rates of 2.1% reported by White et al. [45] and 2.6% by Ahmed et al. [1], although it remains within the 1.26–7.5% range of Tsenkov and Dimitrov [42]. We attribute this to our inclusion of transient neuritis and of events following concomitant ulnar nerve decompression, which was performed in 24% to 36% of patients and which many series exclude. The open OCA ulnar nerve injury rate of 16.9% should be interpreted in the same light.

The magnitude of motion recovery in our OCA groups aligns with prior reports. Antuña et al. documented sustained arc gains after open ulnohumeral arthroplasty at long-term follow-up [2]. Galle et al. reported meaningful improvements after arthroscopic OCA [13]. Kim et al. in a systematic review of 634 elbows, found that open OCA produced somewhat greater flexion and total arc gain than the arthroscopic approach [19], which is consistent with the direction of our overlap-weighted estimates, where open OCA recovered marginally more motion than arthroscopic OCA (35.9% versus 32.2%) despite the matched analysis favouring arthroscopy. The TEA group achieved a two year arc of 94.0° in the overlap population, consistent with Schoch et al., who noted that TEA reliably alleviates pain but inconsistently restores extension [38].

Why did TEA restore less motion? The explanation is partly a matter of indication, TEA patients began with more advanced disease and lower preoperative arcs, and partly mechanical, as the linked semiconstrained implant design inherently constrains terminal flexion and extension [14]. Overlap weighting addresses the first of these by restricting inference to patients whose baseline characteristics were compatible with either strategy; the persistence of the difference within that population suggests a genuine mechanical contribution. Prior literature has noted similar findings [34, 38].

The arthroscopic approach offered a cost advantage over open OCA. Although arthroscopic procedures often carry higher equipment costs, in this cohort the dominant cost drivers were length of stay (4.8 versus 6.8 days) and lower ward, nursing, and materials charges. White et al. found comparable functional outcomes with slightly lower complication rates for arthroscopy in their systematic review of 42 studies [45]. However, arthroscopic elbow surgery demands advanced technical skill, carries a defined learning curve, and can produce meaningful neurovascular complications [1, 18, 42]; our arthroscopic results, obtained by experienced surgeons at high-volume centres, may not be reproducible in lower-volume settings.

Several limitations warrant emphasis. First, radiographic disease severity was not uniformly available across the three centres’ retrospective records, and we could not grade osteoarthritis using validated systems [6, 22, 36]. Because treatment selection is strongly influenced by radiographic severity, unmeasured differences in joint destruction may persist despite weighting; this is the principal residual confounder. Second, the overlap population is a statistical construct, and although it corresponds conceptually to clinical equipoise, it is defined by measured covariates only; patients alike on the recorded variables may still have differed on unrecorded ones such as cartilage loss. Third, an effective sample size of 246 reduces precision relative to the nominal 542, and the TEA contribution of 41 patients is modest. Fourth, the retrospective design introduces selection bias that weighting can reduce but not eliminate. Fifth, medical coding accuracy in Chinese hospitals is variable, with documented error rates of 4.79% to 73.08% [30, 35]; we combined automated code-based extraction with manual chart-review validation of every eligibility criterion and outcome in a 10% random subsample at each site. Sixth, cost data were limited to direct medical costs from a healthcare-system perspective and no utility data were collected, precluding cost-utility analysis. Seventh, follow-up of twom to seven years leaves longer-term TEA implant survivorship undetermined. Eighth, absolute costs and length-of-stay patterns reflect Chinese tertiary practice and may not transfer to other health systems, though the relative ordering of the strategies is likely more portable.

In conclusion, among patients with primary elbow OA for whom joint preservation and arthroplasty were both plausible options, osteocapsular arthroplasty recovered more motion than total elbow arthroplasty at roughly a third of the cost, while pain relief, the objective for which arthroplasty is principally undertaken, was clinically equivalent across all three strategies. These findings describe real-world strategies applied to their usual indications within a defined equipoise sub-population, and they do not displace TEA for patients with end-stage ulnohumeral destruction, who were under-represented in our overlap population. Where the choice is genuinely open, joint-preserving surgery appears the more efficient option; confirmation requires prospective study with radiographic severity grading and preference-based utility measurement.

## Supplementary Information

Below is the link to the electronic supplementary material.

ESM 1 (DOCX 104 KB)

## Data Availability

The datasets generated and analyzed during the current study are not publicly available because they contain sensitive patient-level electronic health record data from Chinese hospitals, the release of which is restricted by the data governance policies of the participating institutions and the Personal Information Protection Law of the People’s Republic of China. De-identified, aggregated summary-level data sufficient to reproduce the main analyses reported in this study are provided in the Supplementary Information. Requests for access to patient-level data for the purpose of reproducing or extending this study can be directed to the corresponding author (Shengdi Lu, Shengdi.Lu@pbrc.edu).

## Declarations

### Reporting guideline compliance

This study was designed, conducted, and reported in accordance with the Strengthening the Reporting of Observational Studies in Epidemiology (STROBE) guidelines for cohort studies. A completed STROBE checklist is available as supplementary material.
