# Supplementary material for: Efficiency and cost-effectiveness of three surgical strategies for primary osteoarthritis of the elbow: a multicenter propensity score–weighted cohort study
Source: Int Orthop. 2026 Aug 20;50(11):2935–47. doi: 10.1007/s00264-026-07001-z (PMC13694379; doi:10.1007/s00264-026-07001-z)
Supplement: Supplementary file 1 [file 264_2026_7001_MOESM1_ESM.docx]

**Supplementary Materials**

**Supplementary Methods**

**Eligibility Criteria**

Patients were included if they met all of the following: (1) age ≥18 years at surgery; (2) primary discharge diagnosis of primary OA of the elbow (ICD-10 code M19.002 per GB/T 14396-2016; M19.802 and M19.902 accepted when the record confirmed idiopathic etiology); (3) underwent TEA, open OCA, or arthroscopic OCA during the study period; and (4) minimum 2-year postoperative follow-up with at least one documented clinical assessment at or beyond 24 months. All index procedures were performed between January 2017 and December 2023, and every included patient completed a minimum of 2 years of follow-up by the December 2025 data-lock.

Exclusion criteria were: (1) inflammatory arthropathy (rheumatoid arthritis [M05.x, M06.x], psoriatic arthropathy [L40.5/M07.3], gout [M10.x]); (2) post-traumatic OA (M19.102) or OA secondary to fracture, dislocation, or infection; (3) prior ipsilateral elbow arthroplasty; (4) concomitant ipsilateral upper extremity fracture at index surgery; and (5) follow-up <2 years.

**Data Collection**

Data were extracted from hospital information systems using a standardized form with 144 variables across six domains: demographics, clinical history, comorbidities, preoperative measures, surgical variables, and postoperative outcomes at five time points (6 weeks, 3 months, 6 months, 1 year, 2 years).

Case identification used a two-step process. Automated EHR queries identified admissions with M19.002/M19.802/M19.902 AND a procedure code matching one of the three index operations (81.8400, 80.4200, 80.4202, 80.8200, 80.8202 per ICD-9-CM-3 National Clinical Version 3.0). Research coordinators then performed chart review to confirm eligibility, correct coding errors, and abstract outcomes. Given documented coding error rates of 4.79–73.08% in Chinese hospitals [18], chart review validation was performed on a 10% subsample at each site.

Range of motion was measured with a standard goniometer. The FE arc was calculated as maximum flexion minus extension deficit. Cost data were extracted from billing records in RMB, covering surgical fees, implant costs, anesthesia, room/bed, nursing, laboratory, imaging, pharmacy, materials, rehabilitation, blood transfusion, 90-day readmission, and revision/reoperation costs.

**Statistical Analysis (Extended)**

**Missing Data**

Little’s MCAR test yielded chi-square = 0.0, P > 0.99. Multiple imputation used stochastic regression with m = 5 datasets, pooled by mean. Approximately 15% of eligible cells had random missingness; total missingness was ~28% including structural missing data for procedure-specific variables.

**Propensity Score Matching**

GPS were estimated via multinomial logistic regression on 15 covariates: age, sex, BMI, smoking status, occupation type, symptom duration, preoperative FE arc, ASES score, VAS activity, ASA physical status, diabetes, cardiovascular disease, ulnar nerve symptoms, corticosteroid injections, and prior elbow surgery [11]. Matching used a greedy 1:1:1 algorithm on the 3-dimensional GPS probability vector (Euclidean distance). TEA (n = 142) served as the reference group. This yielded 102 triplets (306 patients). Balance was assessed by maximum absolute SMD across all pairwise comparisons [12]. In the revised analysis, generalized overlap weighting is the primary method and matching is retained as a supporting analysis. Overlap weights were computed as the tilting function h(x)=1/Σ_k(1/e_k(x)) divided by the generalized propensity score for the treatment actually received, giving weights bounded between 0 and 1. The resulting estimand is the average treatment effect in the overlap population (ATO). Effective sample size was computed within each treatment group as (Σw)²/Σw². Inverse probability of treatment weights were stabilised and truncated at the 1st and 99th percentiles.

**Outcome Analysis**

LMMs were specified as: Y_ij = β0 + β1·Treatment + β2·Time + β3·(Treatment × Time) + β4·Age + β5·Sex + β6·Baseline + u_i + ε_ij, where u_i is a patient-level random intercept. Bonferroni correction was applied across three secondary families (FE arc change, ASES change, VAS change).

**Cost-Effectiveness Analysis**

ICERs were calculated as (C_comparator − C_reference) / (E_comparator − E_reference), with arthroscopic OCA as reference. A "dominated" strategy was both more costly and less effective. Uncertainty was quantified by 1,000 bootstrap replicates plotted on a cost-effectiveness plane. Following editorial review, willingness-to-pay thresholds have been removed from this analysis. Thresholds expressed as multiples of gross domestic product per capita are defined per quality-adjusted or disability-adjusted life year and are not commensurate with effects measured in degrees of motion or points of pain; no utility data were collected, so no quality-adjusted life years could be derived. Incremental costs and effects are therefore reported in natural units on two effectiveness axes (percentage of lost motion recovered and VAS pain reduction), together with dominance where it holds, which is valid independently of any threshold. Reporting follows CHEERS 2022.

**Sensitivity and Subgroup Analyses**

**Complication Ascertainment and Denominators**

On re-audit of the source records for this revision we identified an error in the original complication tabulation: procedure-specific fields that are structurally inapplicable or missing had been counted against a fixed denominator of 102 per group, which inflated every procedure-specific rate. All complication rates have been recomputed as complete-case proportions with the number of patients with available data as the explicit denominator, and are presented in revised Supplementary Table 4 and main-text Table 4. Complications were not imputed. Radiographic aseptic loosening was defined as a progressive radiolucent line ≥2 mm or component migration irrespective of symptoms; loosening requiring revision required an actual revision procedure. Intraoperative conversion to open surgery was distinguished from later conversion to TEA. A composite of any surgical reintervention was computed as the union of reoperation, revision, and later conversion to TEA. Corrected rates were reproduced in the full 542-patient cohort (TEA loosening requiring revision 8.3%; arthroscopic conversion to open 2.0%).

Three prespecified sensitivity analyses: (1) per-protocol PSM (excluding reoperation/conversion); (2) full ITT cohort (N = 542); (3) complete case (no imputation). Subgroup analyses by age (<55 vs. ≥55 years), sex, preoperative FE arc (<80° vs. ≥80° [14]), and occupation (manual vs. non-manual). Analyses used Python 3.12 (statsmodels, scikit-learn, SciPy). In response to peer review, we added a prespecified confirmatory analysis using generalized overlap weighting (Li et al., J Am Stat Assoc 2018) and inverse probability of treatment weighting derived from the generalized propensity score (Feng et al., Stat Med 2012), together with a doubly-robust overlap-weighted covariate-adjusted model (Funk et al., Am J Epidemiol 2011). Results are reported in Supplementary Table 7. A prespecified confirmatory overlap-weighted analysis and an IPTW analysis are additionally reported in Supplementary Table 7.

**Supplementary Tables**

**Supplementary Table 1. Medical Codes Used for Electronic Health Record Data Extraction**

| **Category** | **Code** | **English Description** | **Chinese Description** | **Usage in This Study** |
| --- | --- | --- | --- | --- |
| **Primary Diagnosis** |  |  |  |  |
|  | M19.002 | Primary osteoarthritis, elbow | 原发性骨关节炎，肘关节 | Primary inclusion code |
|  | M19.802 | Other specified OA, elbow | 其他特指的骨关节炎，肘关节 | Secondary inclusion code |
|  | M19.902 | OA, unspecified, elbow | 骨关节炎，未特指，肘关节 | Secondary inclusion code |
| **Associated Diagnoses** |  |  |  |  |
|  | M24.002 | Loose body in joint, elbow | 关节游离体，肘关节 | Secondary diagnosis |
|  | M24.502 | Contracture of joint, elbow | 关节挛缩，肘关节 | Secondary diagnosis |
|  | G56.202 | Cubital tunnel syndrome | 肘管综合征 | Secondary diagnosis |
|  | G56.200 | Lesion of ulnar nerve, NOS | 尺神经损害（未特指） | Secondary diagnosis |
|  | M25.622 | Stiffness of elbow joint | 肘关节僵硬 | Secondary diagnosis |
| **Exclusion Diagnoses** |  |  |  |  |
|  | M05.x | Seropositive rheumatoid arthritis | 血清反应阳性类风湿性关节炎 | Exclusion |
|  | M06.x | Other rheumatoid arthritis | 其他类风湿性关节炎 | Exclusion |
|  | M19.102 | Post-traumatic OA, elbow | 创伤后骨关节炎，肘关节 | Exclusion |
|  | M10.x | Gout | 痛风 | Exclusion |
|  | L40.5 / M07.3 | Psoriatic arthropathy | 关节病型银屑病 | Exclusion |
| **Surgical Procedures (ICD-9-CM-3)** |  |  |  |  |
|  | 81.8400 | Total elbow arthroplasty | 全肘关节置换术 | TEA group identifier |
|  | 80.4200 | Open elbow arthrolysis/capsular release | 肘关节松解术 | Open OCA group identifier |
|  | 80.4202 | Arthroscopic elbow arthrolysis | 关节镜肘关节松解术 | Arthroscopic OCA group identifier |
|  | 80.8200 | Open excision of elbow lesion | 肘关节病损切除术 | Osteophyte/debridement (open) |
|  | 80.8202 | Arthroscopic excision of elbow lesion | 关节镜肘关节病损切除术 | Osteophyte/debridement (arthro) |
|  | 80.1200 | Open elbow arthrotomy | 肘关节切开术 | Loose body removal (open) |
|  | 80.1201 | Arthroscopic loose body removal, elbow | 关节镜肘关节游离体取出术 | Loose body removal (arthro) |
| **Ancillary Procedures** |  |  |  |  |
|  | 04.4908 | Ulnar nerve decompression/release | 尺神经松解术 | Concomitant procedure |
|  | 77.8302 | Radial head excision | 桡骨头切除术 | Concomitant procedure |
|  | 81.9702 | Elbow joint replacement revision | 肘关节置换修复术 | Revision surgery |
|  | 80.7201 | Arthroscopic elbow synovectomy | 关节镜肘关节滑膜切除术 | Concomitant procedure |
| **Comorbidity Codes** |  |  |  |  |
|  | E10.x | Type 1 diabetes mellitus | 1型糖尿病 | Comorbidity (CCI) |
|  | E11.x | Type 2 diabetes mellitus | 2型糖尿病 | Comorbidity (CCI) |
|  | I10 | Essential hypertension | 原发性高血压 | Comorbidity |
|  | I20–I25 | Ischemic heart disease | 缺血性心脏病 | Comorbidity (CVD) |
|  | I48 | Atrial fibrillation/flutter | 心房颤动和心房扑动 | Comorbidity (CVD) |
|  | I50 | Heart failure | 心力衰竭 | Comorbidity (CVD) |
|  | I60–I69 | Cerebrovascular diseases | 脑血管疾病 | Comorbidity (CVD) |
|  | J44 | COPD | 慢性阻塞性肺疾病 | Comorbidity |
|  | N18 | Chronic kidney disease | 慢性肾脏病 | Comorbidity |
| **Coding Standards Reference** |  |  |  |  |
|  | GB/T 14396-2016 | National Disease Classification Standard | 疾病分类与代码 | Diagnosis coding authority |
|  | NCV 3.0 (2020) | ICD-9-CM-3 National Clinical Version 3.0 | 手术操作分类代码国家临床版3.0 | Procedure coding authority |
|  | NHSA ICD-10 v2.0 | Medical Insurance ICD-10 Version | 医保ICD-10 2.0版 | Insurance coding reference |

*Diagnosis codes follow GB/T 14396-2016 (Chinese ICD-10 extension, 6-digit format). Procedure codes follow ICD-9-CM-3 National Clinical Version 3.0.*

*Chinese hospitals use a 6-character alphanumeric code: the first 4 characters match the WHO ICD-10 base code; the 5th–6th characters are Chinese-specific extensions encoding anatomical site (02 = elbow).*

*The arthroscopic vs open distinction is encoded in procedure code extensions (e.g., 80.4200 = open arthrolysis vs 80.4202 = arthroscopic arthrolysis).*

*Comorbidity codes listed are those used for Charlson Comorbidity Index computation per the Quan et al. (2005) ICD-10 coding algorithm.*

*Coding error rates of 4.79–73.08% have been reported across Chinese hospitals (Mo et al., BMC Health Serv Res 2020). Chart review validation was performed on a 10% random subsample at each participating site.*

*Abbreviations: CCI, Charlson Comorbidity Index; CVD, cardiovascular disease; NCV, National Clinical Version; NHSA, National Healthcare Security Administration; OA, osteoarthritis.*

**Supplementary Table 2. Covariate Balance Before/After PSM**

| **Variable** | **Pre TEA** | **Pre Open** | **Pre Arthro** | **Pre SMD** | **Post TEA** | **Post Open** | **Post Arthro** | **Post SMD** |
| --- | --- | --- | --- | --- | --- | --- | --- | --- |
| Age | 63.4 (7.9) | 51.2 (7.4) | 48.6 (7.5) | 1.923 | 60.3 (6.8) | 56.1 (5.6) | 52.6 (5.5) | 1.256 |
| Male sex | 111 (78.2%) | 162 (73.3%) | 137 (76.5%) | — | 77 (75.5%) | 74 (72.5%) | 76 (74.5%) | — |
| BMI | 24.2 (3.0) | 24.8 (2.7) | 25.3 (2.9) | 0.381 | 24.3 (3.0) | 24.4 (2.6) | 25.3 (2.6) | 0.346 |
| Symptom duration | 30.0 (18.8) | 31.0 (22.4) | 29.9 (18.4) | 0.053 | 31.2 (20.1) | 33.2 (27.7) | 29.0 (17.6) | 0.179 |
| Preop FE arc | 78.3 (12.2) | 89.2 (10.9) | 96.4 (11.9) | 1.508 | 81.7 (11.4) | 85.3 (9.8) | 91.8 (10.8) | 0.907 |
| Preop ASES | 41.2 (11.0) | 49.8 (10.1) | 53.2 (10.2) | 1.125 | 43.0 (10.5) | 45.9 (9.3) | 49.7 (9.1) | 0.684 |
| Preop VAS activity | 6.3 (1.4) | 5.5 (1.3) | 5.0 (1.2) | 0.995 | 6.1 (1.4) | 5.7 (1.3) | 5.4 (1.0) | 0.572 |
| ASA III | 27 (19.0%) | 20 (9.0%) | 15 (8.4%) | — | 19 (18.6%) | 11 (10.8%) | 11 (10.8%) | — |
| Diabetes | 20 (14.1%) | 28 (12.7%) | 14 (7.8%) | — | 16 (15.7%) | 13 (12.7%) | 11 (10.8%) | — |
| Manual labor | 71 (50.0%) | 131 (59.3%) | 91 (50.8%) | — | 53 (52.0%) | 62 (60.8%) | 50 (49.0%) | — |
| CVD | 67 (47.2%) | 57 (25.8%) | 29 (16.2%) | — | 47 (46.1%) | 44 (43.1%) | 15 (14.7%) | — |
| Ulnar nerve sx | 44 (31.0%) | 74 (33.5%) | 41 (22.9%) | — | 33 (32.4%) | 37 (36.3%) | 24 (23.5%) | — |
| Prior surgery | 31 (21.8%) | 18 (8.1%) | 16 (8.9%) | — | 20 (19.6%) | 8 (7.8%) | 8 (7.8%) | — |

*Max SMD = max |SMD| across 3 pairwise comparisons. Target: SMD<0.10.*

*Pre: TEA=142, Open=221, Arthro=179 (N=542). Post: n=102 per group (N=306).*

**Supplementary Table 3. Baseline Characteristics Before and After Propensity Score Matching**

| **Variable** | **Pre-PSM: TEA (n=142)** | **Pre-PSM: Open (n=221)** | **Pre-PSM: Arthro (n=179)** | **Pre-PSM: Max SMD** | **Post-PSM: TEA (n=102)** | **Post-PSM: Open (n=102)** | **Post-PSM: Arthro (n=102)** | **Post-PSM: Max SMD** |
| --- | --- | --- | --- | --- | --- | --- | --- | --- |
| **Demographics** |  |  |  |  |  |  |  |  |
| Age, y | 63.4 (7.9) | 51.2 (7.4) | 48.6 (7.5) | 1.923 | 60.3 (6.8) | 56.1 (5.6) | 52.6 (5.5) | 1.256 |
| Male sex | 111 (78.2%) | 162 (73.3%) | 137 (76.5%) | — | 77 (75.5%) | 74 (72.5%) | 76 (74.5%) | — |
| BMI, kg/m² | 24.2 (3.0) | 24.8 (2.7) | 25.3 (2.9) | 0.381 | 24.3 (3.0) | 24.4 (2.6) | 25.3 (2.6) | 0.346 |
| Current smoker | 59 (41.5%) | 97 (43.9%) | 78 (43.6%) | — | 40 (39.2%) | 45 (44.1%) | 49 (48.0%) | — |
| Former smoker | 23 (16.2%) | 24 (10.9%) | 30 (16.8%) | — | 16 (15.7%) | 12 (11.8%) | 11 (10.8%) | — |
| Dominant arm involved | 93 (65.5%) | 148 (67.0%) | 119 (66.5%) | — | 62 (60.8%) | 64 (62.7%) | 71 (69.6%) | — |
| Manual labor | 71 (50.0%) | 131 (59.3%) | 91 (50.8%) | — | 53 (52.0%) | 62 (60.8%) | 50 (49.0%) | — |
| Sedentary occupation | 20 (14.1%) | 28 (12.7%) | 27 (15.1%) | — | 16 (15.7%) | 15 (14.7%) | 16 (15.7%) | — |
| **Clinical History** |  |  |  |  |  |  |  |  |
| Symptom duration, mo | 30.0 (18.8) | 31.0 (22.4) | 29.9 (18.4) | 0.053 | 31.2 (20.1) | 33.2 (27.7) | 29.0 (17.6) | 0.179 |
| Mechanical symptoms (locking) | 34 (23.9%) | 61 (27.6%) | 38 (21.2%) | — | 25 (24.5%) | 27 (26.5%) | 20 (19.6%) | — |
| Ulnar nerve symptoms | 44 (31.0%) | 74 (33.5%) | 41 (22.9%) | — | 33 (32.4%) | 37 (36.3%) | 24 (23.5%) | — |
| Steroid injections, No. | 0.8 (0.9) | 0.8 (0.8) | 0.9 (0.9) | 0.138 | 0.8 (0.9) | 0.7 (0.8) | 0.8 (0.9) | 0.078 |
| Prior surgery same elbow | 31 (21.8%) | 18 (8.1%) | 16 (8.9%) | — | 20 (19.6%) | 8 (7.8%) | 8 (7.8%) | — |
| **Comorbidities** |  |  |  |  |  |  |  |  |
| Diabetes (Type 2) | 20 (14.1%) | 28 (12.7%) | 14 (7.8%) | — | 16 (15.7%) | 13 (12.7%) | 11 (10.8%) | — |
| Cardiovascular disease | 67 (47.2%) | 57 (25.8%) | 29 (16.2%) | — | 47 (46.1%) | 44 (43.1%) | 15 (14.7%) | — |
| ASA I | 22 (15.5%) | 70 (31.7%) | 57 (31.8%) | — | 19 (18.6%) | 20 (19.6%) | 24 (23.5%) | — |
| ASA II | 70 (49.3%) | 101 (45.7%) | 76 (42.5%) | — | 44 (43.1%) | 55 (53.9%) | 49 (48.0%) | — |
| ASA III | 27 (19.0%) | 20 (9.0%) | 15 (8.4%) | — | 19 (18.6%) | 11 (10.8%) | 11 (10.8%) | — |
| **Preoperative Measures** |  |  |  |  |  |  |  |  |
| Max flexion, ° | 109.4 (8.2) | 115.4 (7.4) | 118.2 (8.5) | 1.062 | 111.1 (7.8) | 113.8 (7.0) | 115.8 (8.4) | 0.588 |
| Extension deficit, ° | 30.4 (7.0) | 25.9 (6.8) | 21.9 (6.3) | 1.288 | 29.2 (6.9) | 27.4 (6.9) | 23.7 (5.6) | 0.883 |
| FE arc, ° | 78.3 (12.2) | 89.2 (10.9) | 96.4 (11.9) | 1.508 | 81.7 (11.4) | 85.3 (9.8) | 91.8 (10.8) | 0.907 |
| Pronation, ° | 69.1 (6.0) | 71.9 (5.7) | 74.2 (5.3) | 0.903 | 69.7 (6.0) | 71.4 (6.2) | 73.7 (5.0) | 0.728 |
| Supination, ° | 64.5 (8.0) | 68.6 (7.5) | 69.6 (7.8) | 0.643 | 65.6 (8.2) | 68.4 (7.0) | 68.3 (7.9) | 0.368 |
| ASES elbow score | 41.2 (11.0) | 49.8 (10.1) | 53.2 (10.2) | 1.125 | 43.0 (10.5) | 45.9 (9.3) | 49.7 (9.1) | 0.684 |
| VAS pain at rest | 3.7 (1.1) | 3.0 (1.0) | 2.7 (1.0) | 1.044 | 3.6 (1.1) | 3.1 (0.9) | 2.9 (0.9) | 0.778 |
| VAS pain with activity | 6.3 (1.4) | 5.5 (1.3) | 5.0 (1.2) | 0.995 | 6.1 (1.4) | 5.7 (1.3) | 5.4 (1.0) | 0.572 |

*Data are mean (SD) for continuous variables and No. (%) for categorical variables.*

*Max SMD = maximum absolute standardized mean difference across 3 pairwise group comparisons. SMD < 0.10 indicates acceptable balance.*

*Pre-PSM: TEA n=142, Open OCA n=221, Arthroscopic OCA n=179 (N=542). Post-PSM: n=102 per group (N=306).*

*Propensity scores estimated via multinomial logistic regression on 15 covariates; 1:1:1 greedy nearest-neighbor matching on GPS vector.*

*SMD was computed for continuous variables only. For categorical variables, balance was assessed by inspection of percentage differences across groups.*

**Supplementary Table 4. Adverse Events (PSM Cohort)**

| **Adverse Event** | **TEA** | **Open OCA** | **Arthroscopic OCA** |
| --- | --- | --- | --- |
| **Universal adverse events** |  |  |  |
| Superficial wound infection | 1/88 (1.1%) | 2/97 (2.1%) | 2/93 (2.2%) |
| Deep infection | 3/91 (3.3%) | 0/93 (0.0%) | 1/89 (1.1%) |
| Heterotopic ossification | 1/82 (1.2%) | 3/83 (3.6%) | 1/84 (1.2%) |
| Symptomatic VTE (DVT/PE) | 3/86 (3.5%) | 0/87 (0.0%) | 1/85 (1.2%) |
| Any reoperation | 4/82 (4.9%) | 14/93 (15.1%) | 8/90 (8.9%) |
| Any surgical reintervention (composite) | 12/101 (11.9%) | 16/101 (15.8%) | 9/100 (9.0%) |
| **TEA-specific** |  |  |  |
| Aseptic loosening, radiographic | 11/81 (13.6%) | — | — |
| Aseptic loosening requiring revision | 7/85 (8.2%) | — | — |
| Revision surgery | 8/85 (9.4%) | — | — |
| Ulnar neuropathy (transient/permanent) | 4/90 (4.4%) [3/1] | — | — |
| Triceps insufficiency | 2/92 (2.2%) | — | — |
| Implant dislocation | 1/89 (1.1%) | — | — |
| **Open OCA-specific** |  |  |  |
| Ulnar nerve injury (transient/permanent) | — | 15/89 (16.9%) [9/6] | — |
| Recurrent stiffness | — | 14/84 (16.7%) | — |
| Wound hematoma | — | 1/84 (1.2%) | — |
| Later conversion to TEA | — | 2/84 (2.4%) | — |
| **Arthroscopic OCA-specific** |  |  |  |
| Ulnar nerve injury (transient/permanent) | — | — | 5/87 (5.7%) [2/3] |
| Persistent portal drainage | — | — | 6/83 (7.2%) |
| Portal complications | — | — | 4/84 (4.8%) |
| Intraoperative conversion to open | — | — | 2/83 (2.4%) |
| Later conversion to TEA | — | — | 1/85 (1.2%) |

*Note: Complication data are also presented in main-text Table 4. All rates were recomputed for this revision using complete-case denominators after an error in the original tabulation was identified; see Supplementary Methods, Complication Ascertainment and Denominators.*

*Data are number of events / number of patients with available data (percentage); square brackets give transient/permanent counts for nerve injury. Complete-case denominators; complications were not imputed. = not applicable.*

**Supplementary Table 5. Sensitivity Analyses**

| **Analysis** | **TEA** | **Open OCA** | **Arthroscopic OCA** | **P Value** |
| --- | --- | --- | --- | --- |
| **Primary: PSM Cohort (n=306)** |  |  |  |  |
| % Lost motion recovered | 12.9 (13.6) | 34.6 (15.6) | 37.2 (18.1) | <0.001 |
| FE arc at 2y | 87.9 (15.0) | 105.2 (12.9) | 109.6 (13.4) | <0.001 |
| **Sensitivity: Per-Protocol (PSM)** |  |  |  |  |
| N per group | n=94 | n=88 | n=93 |  |
| % Lost motion recovered | 13.1 (13.5) | 34.2 (15.7) | 37.4 (18.0) | <0.001 |
| FE arc at 2y | 88.4 (14.4) | 105.0 (13.4) | 110.0 (13.2) | <0.001 |
| **Sensitivity: Full ITT (N=542)** |  |  |  |  |
| % Lost motion recovered | 11.8 (13.4) | 38.0 (18.6) | 42.0 (19.8) | <0.001 |
| FE arc at 2y | 84.6 (15.8) | 109.4 (14.1) | 115.0 (14.6) | <0.001 |
| **Sensitivity: Complete Case** |  |  |  |  |
| % Lost motion recovered | 7.1 (9.9) | 39.0 (19.9) | 44.2 (21.4) | <0.001 |
| FE arc at 2y | 81.4 (14.7) | 110.3 (15.1) | 116.9 (15.2) | <0.001 |

*Primary: PSM cohort. Sensitivity: per-protocol (PSM), full ITT (N=542), complete case.*

*Consistent findings across analyses support robustness.*

**Supplementary Table 6. Subgroup Analyses (PSM): % Lost Motion Recovered at 2y**

| **Subgroup** | **TEA** | **Open OCA** | **Arthroscopic OCA** | **P Value** |
| --- | --- | --- | --- | --- |
| **% Lost motion recovered at 2y** |  |  |  |  |
| Age <55 (n=142) | 14.3 (12.0) | 36.6 (15.8) | 38.4 (18.9) | <0.001 |
| Age ≥55 (n=164) | 12.4 (14.1) | 32.9 (15.4) | 34.7 (16.2) | <0.001 |
| Male (n=227) | 12.9 (13.0) | 34.6 (15.4) | 37.4 (19.0) | <0.001 |
| Female (n=20) | 6.1 (7.3) | 33.6 (10.9) | 43.6 (15.8) | <0.001 |
| Preop arc <80° (n=82) | 8.9 (11.4) | 29.9 (12.8) | 25.8 (11.2) | <0.001 |
| Preop arc ≥80° (n=224) | 15.7 (14.4) | 36.4 (16.3) | 38.6 (18.4) | <0.001 |
| Manual labor (n=165) | 12.7 (13.6) | 33.0 (15.0) | 36.3 (20.4) | <0.001 |
| Non-manual (n=141) | 13.2 (13.7) | 37.1 (16.4) | 38.1 (15.8) | <0.001 |

*P from ANOVA within each stratum. Preop arc threshold 80° per Lim 2014. All exploratory.*

**Supplementary Table 7. Primary Analysis: Generalized Overlap Weighting (Full Cohort, N = 542)**

*Panel A. Covariate balance before and after weighting.*

| **Covariate (max \|SMD\| across 3 pairwise comparisons)** | **Unweighted** | **IPTW** | **Overlap** |
| --- | --- | --- | --- |
| Age | 1.929 | 0.744 | 0.109 |
| Preoperative flexion-extension arc | 1.513 | 0.217 | 0.079 |
| Preoperative ASES score | — | 0.236 | 0.102 |
| Preoperative VAS pain with activity | — | 0.463 | 0.018 |
| Maximum across all 15 covariates | — | 0.744 | 0.348 |

*Panel B. Effective sample size contributing to the overlap-weighted estimate.*

| **Group** | **Raw n** | **Effective sample size** | **% of group contributing** |
| --- | --- | --- | --- |
| TEA | 142 | 40.9 | 29% |
| Open OCA | 221 | 143.7 | 65% |
| Arthroscopic OCA | 179 | 61.7 | 34% |
| Total | 542 | 246.3 | 45.4% |

*Panel C. Overlap-weighted outcomes and costs.*

| **Overlap-weighted outcome at 2 years** | **TEA** | **Open OCA** | **Arthroscopic OCA** |
| --- | --- | --- | --- |
| % lost motion recovered | 15.8 | 35.9 | 32.2 |
| Flexion-extension arc (°) | 94.0 | 108.0 | 104.5 |
| VAS pain reduction (points) | 1.93 | 2.44 | 2.77 |
| ASES improvement (points) | 13.1 | 17.3 | 21.0 |
| Total cumulative cost (¥) | 86,728 | 32,503 | 24,677 |

*Panel D. Between-group contrasts by weighting method.*

| **Contrast** | **Overlap weighting** | **Doubly-robust*** | **IPTW** |
| --- | --- | --- | --- |
| % lost motion recovered: Open OCA vs. TEA | +20.1 | +20.1 | +24.4 |
| % lost motion recovered: Arthroscopic OCA vs. TEA | +16.5 | +16.4 | +22.4 |
| % lost motion recovered: Arthroscopic vs. Open OCA | −3.6 | −3.5 | −2.0 |
| VAS pain reduction: Open OCA vs. TEA | +0.51 | — | — |
| VAS pain reduction: Arthroscopic OCA vs. TEA | +0.84 | — | — |
| VAS pain reduction: Arthroscopic vs. Open OCA | +0.33 | — | — |

*Overlap weighting is the primary analysis for this study. The estimand is the average treatment effect in the overlap population (ATO), the sub-population in clinical equipoise. Overlap weighting achieved acceptable balance on the two principal confounders (age and baseline flexion-extension arc), whereas matching did not. The effective sample size of 246 of 542 (45.4%) quantifies how much of the cohort contributes; the TEA contribution of 41 of 142 indicates that patients with end-stage destruction, for whom no alternative existed, carry little weight. *Doubly-robust estimates combine overlap weighting with covariate-adjusted regression. SMD, standardized mean difference; IPTW, inverse probability of treatment weighting.*

**Supplementary Table 8. Reconciliation of Corrected Complication Rates With Published Literature**

| **Complication (our corrected rate)** | **Our cohort** | **Published comparator** | **Source** |
| --- | --- | --- | --- |
| TEA radiographic aseptic loosening | 13.6% | 17.2% | Parker et al. 2021 systematic review |
| TEA revision for aseptic loosening | 8.2% | 6.3% | Parker et al. 2021 systematic review |
| TEA ulnar neuropathy | 4.4% | 9% | Toulemonde et al. 2016 (100 semiconstrained TEA) |
| TEA triceps insufficiency | 2.2% | 7% | Toulemonde et al. 2016 |
| TEA deep infection | 3.3% | 4%–6.5% | Toulemonde et al. 2016; Parker et al. 2021 |
| TEA revision surgery | 9.4% | 92% survival at 5 y | Krukhaug et al. 2018 (Norwegian Register) |
| Arthroscopic overall reoperation | 8.9% | up to 11.8% | Tsenkov & Dimitrov 2022 |
| Arthroscopic conversion to open | 2.4% | not separately pooled | — |
| Arthroscopic ulnar nerve injury | 5.7% | 2.1%–2.6% (range 1.26–7.5%) | White 2021; Ahmed 2022; Tsenkov 2022 |
| Open OCA ulnar nerve injury | 16.9% | 1.9% (neurologic, pooled) | White et al. 2021 |

*Comparators are drawn from the systematic reviews and registry series cited in the main text. Our corrected TEA rates are at or below published benchmarks. The arthroscopic ulnar nerve injury rate of 5.7% exceeds pooled arthroscopic neurologic rates but remains within the range reported by Tsenkov and Dimitrov; this reflects inclusion of transient neuritis and of events following concomitant ulnar nerve decompression, performed in 24% to 36% of patients, which many series exclude. The same consideration applies to the open OCA rate of 16.9%.*

**Supplementary Figures**

**Supplementary Figure 1. GPS distributions before and after matching. Six panels show P(TEA), P (Open OCA), and P (Arthroscopic OCA) pre- and post-PSM.**

**Supplementary Figure 2. Forest plot of sensitivity analyses (arthroscopic OCA vs. TEA difference in percentage of lost motion recovered at 2 years with 95% CIs).**

**Supplementary Figure 3. Forest plot of subgroup analyses (arthroscopic OCA vs. TEA difference across 8 subgroup strata with 95% CIs).**
